# A Methanogenic Consortium Was Active and Exhibited Long-Term Survival in an Extremely Acidified Thermophilic Bioreactor

**DOI:** 10.3389/fmicb.2019.02757

**Published:** 2019-11-26

**Authors:** Wenhao Han, Pinjing He, Yucheng Lin, Liming Shao, Fan Lü

**Affiliations:** ^1^State Key Laboratory of Pollution Control and Resource Reuse, Tongji University, Shanghai, China; ^2^Shanghai Institute of Pollution Control and Ecological Security, Shanghai, China; ^3^Institute of Waste Treatment and Reclamation, Tongji University, Shanghai, China

**Keywords:** thermophilic anaerobic digestion, extreme acidification, meta-omics, long-term survival, transcriptional regulation, acid tolerance, self-adaptation, stress responses

## Abstract

Acid crisis characterized by acid accumulation and/or low pH is a common reason for the failure of anaerobic digestion (AD), which is usually applied for wastewater and waste treatment. Acid-tolerant methanogens are rarely reported to be active in the artificial anaerobic digester. In this study, we observed that the thermophilic methanogenesis by a consortium in the form of flocs and not granules could still be recovered during long-term operation at acetate concentration of up to 104 mM and pH 5.5 by adjusting the pH gradually or directly to pH 5.5 or 5.0. The acclimation process involving the gradual decrease in pH could enhance the resistance of the consortium against extreme acidification. The stable isotopic signature analysis of biogas revealed that *Methanosarcina*, which produced methane through acetoclastic methanogenesis (AM) pathway, was the predominant methane producer when the pH was decreased gradually to 5.0. Meanwhile, the abundance of *Coprothermobacter* increased with a decrease in pH. Contrastingly, when directly subjected to an environment of pH 5.5 and 104 mM acetate (15.84-mM free acetic acid) after a 42-day lag phase, *Methanothermobacter* was the predominant methanogen. *Methanothermobacter* initiated methane production through the hydrogenotrophic pathway and formed syntrophic relationship/consortium with the potential acetate-oxidizing bacteria, *Thermacetogenium* and *Coprothermobacter*. Comparative metagenomic and metatranscriptomic analysis on this self-adapted and acid-tolerant consortium revealed that the genes, such as GroEL, DnaK, CheY, and flagellum-related genes (FlaA, FlgE, and FliC) from *Anaerobaculum*, *Thermacetogenium*, and *Coprothermobacter* were highly overexpressed in response to system acidification. Microbial self-adaptation patterns (community structure adjustment, methanogenesis pathway shift, and transcriptional regulation) of thermophilic methanogenic consortium to gradual and sudden acidification were evaluated by integrated stable isotopic signature and comparative meta-omic approaches. The study elucidated the acid-resistant mechanism of thermophilic methanogenic consortium and deepened our knowledge of the function, interaction, and microbial characteristics of *Methanosarcina*, *Methanothermobacter*, and *Coprothermobacter* under extreme acidic environment.

## Introduction

Biogas production by AD is a sustainable solution for wastewater treatment and energy recovery from waste (Oosterkamp et al., 2016; Puyol et al., 2016; Calusinska et al., 2018). The mesophilic AD is performed at approximately 37°C, while the thermophilic AD is performed at approximately 55°C. Thus, the thermophilic AD is associated with higherhydrolytic activities and methane recovery and shorter hydraulic retention time than the mesophilic AD (Labatut et al., 2014; Wilkins et al., 2015; Jang et al., 2016). However, there are several limitations of the thermophilic AD system, such as poor system stability, unsatisfactory effluent quality, and high microbial susceptibility to inhibitory compounds (Ghasimi et al., 2015; Gaby et al., 2017; Lü et al., 2018). The limitations associated with thermophilic AD include the accumulation of VFAs, low pH induced by enhanced hydrolysis, and inhibition of methanogenesis due to acidification, which usually results in the failure of the whole system (Joyce et al., 2018; Li et al., 2018).

Although most methanogens grow optimally under neutral conditions circumstance (Demirel and Scherer, 2008), some acidophilic or acid-tolerant methanogenic strains were reported to survive under extremely acidic natural environments. These strains have evolved and acclimated to the low pH condition over several 1000 years and are usually mesophilic. These mesophilic strains include hydrogenotrophic *Methanoregula boonei* 6A8 (pH 4.0–5.8, <200 μM acetate concentration in the medium) (Brauer et al., 2006) or 6A8^*T*^ (pH = 4.0–4.5, completely inhibited with 5 mM acetate) (Brauer et al., 2011), *Methanobacteriales* sp. 26-5a1/*Methanomicrobiales* K-4a2/Rice-Cluster-I K-5a2 (pH 4.0–6.0, acetate <5 mM) (Sizova et al., 2003), *Methanobacterium* sp. MB4 (pH 4.5, acidophilic and psychrotolerant, 0.5 mM acetate) (Kotsyurbenko et al., 2007). All these strains are found in the natural acidic peat bogs and are mesophilic and hydrogenotrophic. However, these acid-tolerant methanogens, except for granular consortia, are rarely detected in the artificial anaerobic digesters, which has an industrial application history of only a few decades. Notably, the methanogens in the core of a granular matrix could be well protected by the surrounding bacteria in the outer layer and hence are not directly exposed to the acidic environment (Sekiguchi et al., 1999; Ishii et al., 2005).

Although highly acidophilic methanogens are not known, previous studies have reported that the methanogenic consortia in some anaerobic bioreactors adapt to the acidic environment with high organic loading. Lins et al. (2014) demonstrated that the high acetate load (pH 7.5, 150 mM acetate) was mitigated in the thermophilic AD by the activity of the robust acetoclastic genus, *Methanosarcina*. Mosbaek et al. (2016) also reported that *Methanosarcina* and *Methanoculleus* along with *Clostridia* were actively involved in acetate turnover at pH 7.64 and acetate concentration of up to 100 mM and probably produced methane through SAO coupled with HM. Hao et al. (2011) also demonstrated the predominant contribution of the SAO pathway to thermophilic acetate methanation at high acetate concentrations (pH 6.8–7.8, 100 mM acetate). In another study, stepwise increased concentration of approximately 7.8, acetate) was fed to the thermophilic AD reactor, which shifted the dominant acetate conversion pathway from SAO-HM to AM (Fotidis et al., 2013; Franke-Whittle et al., 2014). Hori et al. (2015) (pH 7.1, up to about 380 mM acetate) and Akuzawa et al. (2011) (pH 6.1–7.1, <1.7–52 mM acetate) elucidated the structural reorganization of the bacterial and archaeal populations in response to acidification of thermophilic anaerobic digester. The methanogenic consortium is markedly inhibited at low pH value as low pH can increase the concentration of free acid molecules, which are membrane-permeant and more harmful to the microbes than the ionic state. Additionally, low pH can also affect enzymatic activities. Hao et al. (2012) demonstrated that the microbial community structure and methanogenic pathways changed when biogas-bio production recovered from a sudden low pH and high acetate crisis (pH 5.5–6.5, 100 mM acetate, methanogenesis was completely inhibited at pH 5.0) in the thermophilic biogas reactors. Although the prevalence of relevant microbial populations is well understood, the mechanism underlying the microbial ecological “adjustment” is still unclear. Further, the mechanism underlying the response of consortia to the gradual and sudden acidification is unclear during long-term operation. Moreover, it is also not known if the methanogens can survive under extremely acidic environment with high concentration of free acidic molecules (pH below 5.0 and organic loading up to 100 mM acetate) or even recover/preserve the ability for methane production.

Anaerobic digestion with high organic loading is susceptible to failure due to varying degrees of acidification and low pH. Although there are several studies on stress resulting from high organic loading or low pH, very little is known about the methanogenesis performance and microbial characteristics of the consortium upon simultaneous exposure exposed to high organic loading and low pH. In this study, we set up the artificial thermophilic anaerobic digesters with high acetate concentration (104 mM) and decreased the pH gradually or directly to extremely low pH (pH 5.0) to simulate different patterns of acidification. The response of the microbial communities to both acidifying strategies was investigated. Biogas stable carbon isotope-based analytical techniques, 16S rRNA amplicon sequencing, and comparative analysis of the metagenome and meta transcriptome were used to understand the methanogenesis pathway, microbial community structure and metabolic characteristics of the consortium exposed to acid crisis.

## Materials and Methods

### Set Up of Reactors With Different pH Regulating Strategies

**FIGURE 1:**
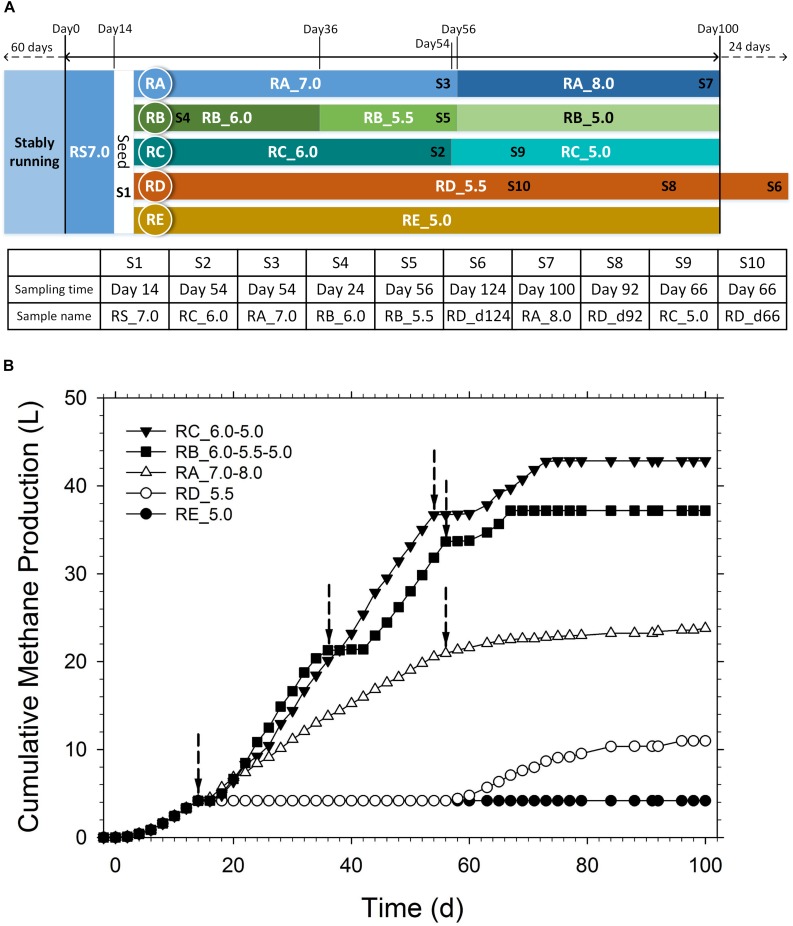
**(A)** Diagram of the pH regulation schedule and biomass sampling points. **(B)** Cumulative methane production under different pH values during the operation period. The arrows indicate the time points for pH adjustment.

The methanogenic sludge flocs were used as inoculum in a 16-L anaerobic sequenced batch reactor (ASBR; marked as RS 7.0, details of the reactor are provided in Supplementary Section 1) inoculum. Briefly, the ASBR was operated at 55°C with acetate as the substrate. The concentration of acetate was maintained at 104 mM every 2 days. The harvested sludge was redistributed into five 1.2-L ASBRs (RA, RB, RC, RD, and RE) with each reactor holding 1.0 L of medium containing 5 g of VSS (volatile suspended solid)/L seed inoculum. As illustrated in Figure 1A, The RA reactor was operated at pH 7.0 initially, which was later changed to pH 8.0. The RB reactor was operated at pH 6.0 initially, which was then changed to pH 5.5, followed by pH 5.0. The RC reactor was operated at pH 6.0 initially, which then changed to pH 5.0. The RD and RE reactors were maintained at pH 5.5 and 5.0 throughout, respectively.

The temperature of the reactors was maintained at 55°C by water bath. Each cycle of the reactor operation included substrate filling, anaerobic reaction in a batch mode, floc settlement, and effluent discharge, which took 2 days. Briefly, the ASBR was filled with 0.1 L of fresh medium containing acetate and micronutrients (Supplementary Section 1), The pH was adjusted to the set values with 3MNaOH or HCl solutions. At the beginning of each reaction cycle, acetate was added to ensure that the final concentration of acetate in each ASBR was 104 mM, which similar to the acetate concentration in the RS 7RS7.0 reactor. The dosage was determined according to the detected acetate concentration at the end of the previous cycle. After the anaerobic reaction step, the stirrer was stopped to allow the sludge flocs to settle. Next, 0.1 L of the supernatant was discharged. Gas, liquid, and liquid-solid mixture samples were collected for further analyses.

### Physio-Chemical Analyses of Gas and Liquid Samples

The gas components (H_2_, CH_4_, and CO_2_) were measured by gas chromatography (GC112A; Shanghai Precision and Scientific Instrument Co., Ltd, Shanghai, China) equipped with a flame ionization detector and thermal conductivity detector. The volume of the gas was determined using a gas meter (TG05/6; Ritter, Bochum, Germany). The stable carbon isotopic compositions of the produced CH_4_ (δ^13^CH_4_) and CO_2_ (δ^13^CO_2_) were periodically monitored using isotope ratio mass spectrometry (Delta V Advantage; Thermo Electron Corporation, United States) linked to gas chromatography (6980N; Agilent Technologies, Santa Clara, CA, United States). The CO_2_ gas standard (δ^13^C_*VPDB*_ = –27.5‰) was injected before and after each gas analysis. The pH of the liquid samples was determined using a pH meter (PXSJ-216F; Shanghai Precision and Scientific Instrument Co., Ltd., Shanghai, China). Subsequently, the samples were centrifuged at 4,460 *g* and 4°C for 10 min using a high-speed refrigerated centrifuge (TL-18 M; Shanghai Centrifugal Machinery Research Institute, Shanghai, China). The supernatant was collected for further analyses. Dissolved organic carbon (DOC) and inorganic carbon were measured using a Total Carbon/Total Nitrogen analyzer (TOC-VCPN, TNM-1, Shimadzu, Kyoto, Japan).

### Data Processing of Stable Carbon Isotope Signatures

In the current system, acetate served as the sole organic carbon source. Thus, it was assumed that methane was mainly produced via AM or HM pathways (methane was unlikely to be produced by methylotrophic methanogenesis, which can only utilize substrates, such as methanol, methylamine, and dimethyl sulfate substrates). The isotope value of the produced CH_4_ (δ^13^CH_4_) was defined as shown in Eq. 1. δ*_ma_* and δ*_mh_* are the isotope values of the CH_4_ produced from AM and HM, respectively. *f*_mh_ is defined as the fraction of CH_4_ produced by HM and was calculated using Eq. 2.

(1) $$\delta^{13}\,{\mathrm{CH}}_{4}=f_{\mathrm{mh}}\cdot \delta_{\mathrm{mh}}+\left(1-f_{\mathrm{mh}}\right)\cdot \delta_{\mathrm{ma}}$$

Thus,

(2) $$f_{\mathrm{mh}}=\left(\delta^{13}\,{\mathrm{CH}}_{4}-\delta_{\mathrm{ma}}\right)/\left(\delta_{\mathrm{mh}}-\delta_{\mathrm{ma}}\right)$$

As δ^13^CH_4_ is a measured variable, the δ*_ma_* and δ*_mh_* values can be calculated from the isotopic signature of their precursors (acetate and CO_2_ produced from acetate in our experiment, δ^13^CH_3_COO^–^ and δ^13^CO_2_) and the fractionation factors of AM and HM (α_ma_ and α_m__h_), respectively (Eqs 3 and 4).

(3) $$\delta_{\mathrm{ma}}=\delta^{13}\,{\mathrm{CH}}_{3}\,{\mathrm{COO}}^{-}+10^{3}\times \left(1-\alpha_{\mathrm{ma}}\right)$$

(4) $$\delta_{\mathrm{mh}}=\delta^{13}\,{\mathrm{CO}}_{2}+10^{3}\times \left(1-\alpha_{\mathrm{mh}}\right)$$

However, data for δ^13^CH_3_COO^–^ are lacking, and the values for α_ma_ and α_m__h_ in the various environments also vary (Conrad, 2005). Thus, it is difficult to precisely determine the values of δ*_ma_* and δ*_mh_* in a mixed culture. In this study, the average δ*_ma_* and α_m__*h*_ values were set at values-33.7‰ and 1.064, respectively, according to the previous reports (Conrad, 2005). δ^13^CO_2_ is a measured variable.

In addition to *f*_mh_, an apparent fractionation factor (α_c_) can also be used to evaluate the predominant methanogenic pathways, which is calculated by the measured δ^13^CH_4_ and δ^13^CO_2_ (Eq. 5). Previous studies have suggested that α_c_ > 1.065 and α_c_ < 1.055 (mostly < 1.027) are characteristics for methanogenesis mediated by the hydrogenotrophic and acetoclastic pathways, respectively (Whiticar et al., 1986; Whiticar, 1999).

(5) $$\alpha_{c}=\left(\delta^{13}\,{\mathrm{CO}}_{2}+1000\right)/\left(\delta^{13}\,{\mathrm{CH}}_{4}+1000\right)$$

### DNA and RNA Extraction and High-Throughput Sequencing

Ten biomass samples (labeled as S1–S10) were collected from the reactors as shown in Figure 1A. Sample S6 from RD_5.5 was used for both DNA and RNA extraction for metagenomic and metatranscriptomic sequencing. The liquid-solid mixtures were vortex-mixed for 1 min and centrifuged at 6,000 *g* and 4°C for 10 min. The cell pellet was used for DNA and RNA extraction. The total DNA in each sample was extracted using the PowerSoil^®^ DNA isolation kit (Mo-Bio Laboratories Inc., CA, United States) following the manufacturer’s instructions. The DNA yields were determined using the SpectraMax 190 system (Molecular Devices, San Jose, CA United States), while the DNA integrity was evaluated via electrophoresis using 1.0% agarose gel. The quality and quantity of total DNA were also estimated using the NanoDrop 2000 spectrophotometer. The 515F (5′-GTGCCAGCMGCCGCGGTAA-3′) and 806R (5′-GGACTACHVGGGTWTCTAAT-3′) primers were used to amplify the V4 region of the microbial 16S rRNA gene by PCR.

Total RNA was extracted using an RNeasy Mini Kit (Qiagen, Hilden, Germany), following the manufacturer’s instructions. The DNA in the RNA sample was removed using an RNase-Free DNase Set (Qiagen, Germany), following the manufacturer’s instructions. The quality and quantity of total RNA produced were estimated using the NanoDrop 2000 spectrophotometer and Agilent 2100 Bioanalyzer (Agilent Technologies, Palo Alto, CA, United States). The cDNA library was prepared for sequencing from the RNA product by reverse transcription using the TruSeq RNA Sample Prep kit (Illumina, San Diego, CA, United States), following the manufacturer’s instructions. During the cDNA preparation and sequencing procedures, during Human UHR total RNA (Agilent Technologies; catalog # 740000) was used as a control to.

Sequencing of the 16S rRNA gene amplicons was performed using an Illumina MiSeq platform (PE250, Illumina, San Diego, CA, United States) at Shanghai Genergy Biotechnology Co., Ltd. (Shanghai, China), The total DNA and cDNA libraries were sequenced using the Illumina HiSeq 2500 platform (Illumina, San Diego, CA, United States) at Majorbio Bio-pharm Technology Co., Ltd., Shanghai, China. The detailed pretreatment and sequencing procedures were performed as described by Wang et al. (2016) and our previous work (Han et al., 2018).

### Bioinformatics and Statistical Analysis

The raw 16S rRNA gene amplicon sequences were firstly trimmed, to remove the sequences with an average quality value of below 20 or with a size less than 20 bp. The high-quality sequences were further analyzed by QIIME 2 (Bokulich et al., 2018) to perform operation taxonomic unit (OTU) clustering with 97% identity threshold. RDP classifier (Wang et al., 2007) was used to annotate the taxonomic information. The PAST (v.3.1.0) software was utilized to perform principal component analysis (PCA) based on the Bray–Curtis distance for total microbiomes. The metagenomic and metatranscriptomic analyses were conducted using the pipeline described in our previous study (Han et al., 2018). Briefly, the metagenomic and metatranscriptomic sequences were annotated against the databases available (including RefSeq, IMG, TrEMBL, SEED, KEGG, and GenBank for taxonomic assignment and Subsystems, COG and KO for functional profiling) on the Metagenomics Rapid Annotation (MG-RAST) server (v4.0)^1^. Additionally, the HUMAnN2 was used to annotate the genome of specific microbial members at the species level (Franzosa et al., 2018).

## Results

### Methanization Performance

The cumulative methane production under various pH values during the operation period is shown in Figure 1B. The average methane production rates at different operation periods are shown in Supplementary Table S1. During the first 14 days when the pH was maintained at 7.0, the average methane production rate was 260.3 mL/d. In the RA reactor, the pH was maintained at 7.0 between days 14 and 56, which increased the average methane production rate to 399.4 mL/d. Next, the pH in the RA reactor was adjusted to 8.0, which sharply decreased the methane production rate (79.5 mL/d). This indicated that methanogenesis was inhibited under alkaline conditions. However, some methanogens still survived and contributed to the slow methane production in the following days.

In the RB and RC reactors, the pH was decreased to 6.0 during days 14–36 and days 14–54, respectively. The average methane production rates in the RB and RC reactors were 778.7 and 813.9 mL/d, respectively, which were twice the rate observed in the RA reactor when the pH was maintained at 7.0. In the RB reactor, the pH was decreased periodically to 5.5 and 5.0 in the following periods. At pH 5.5, the average methane production rate increased to 877.0 mL/d after a short lag phase of 6 days. However, the average methane production rate decreased to 570.0 mL/d when the pH was adjusted to 5.0. There was also a 4-day lag phase without any methane production and the gas production resumed at day 60. However, the methane production lasted only 9 days before methanogenesis activity was completely inhibited. When the pH of the RC reactor was adjusted from 6.0 to 5.0 directly, the methane production immediately stopped and was followed by a 4-day lag phase. The methane production restarted on day 60 and only lasted 14 days. The average methane production rate was 495.1 mL/d, which was higher than that under a neutral environment in the RS and RA reactors. In the RD and RE reactors, the pH was changed from 7.0 to 5.5 and 5.0, respectively. When the pH was maintained at 5.0 in the RE reactor, the extremely acidic environment completely inhibited methanogenesis throughout the operation period. When the pH was maintained at 5.5 in the RD reactor, methanogenesis was inhibited until day 56. However, methanogenesis resumed with the average methane production rate of 189.0 mL/d post-day 56.

Compared to the RA reactor maintained at pH 7.0 at the early stage, the methane production rates increased by 0.0, 47.3, 199.4, and 19.9% when the pH was adjusted to 5.0, 5.5, 6.0, and 8.0, respectively, in the RB and RC reactors. This indicated that methane production improved under weakly acidic conditions (pH 6.0) during the AD of high concentrations of acetic acid. This did not concur with the conventional knowledge that considers neutral pH as optimal. However, the metabolic activity of methanogens would be greatly suppressed at low pH (pH ≤ 5.5). The direct and sudden exposure to the extremely acidic environment inhibits the methanogenesis system or results in a long lag phase. However, the gradual and periodical decrease in pH enables the acid-tolerant methanogens to acclimatize and increase their acid resistance, which results in higher methane production in acidic conditions. The average methane production rates at the three stages in the RB reactor (pH 6.0, 5.5, and 5.0) were higher than those under neutral conditions, although the days of lag phase continued and the recovered methanogenic activities only lasted for a short period (<14 days) (Supplementary Table S1). Therefore, the methanization system could not be operated stably in the long-term under extremely acidic conditions (pH 5.0) even with proper acclimation. It would be interesting to evaluate the microbial ecology during this procedure, especially the methanogens and methanogenic pathways.

### Methanogenic Pathways Evaluated by Stable Carbon Isotope Signatures

**FIGURE 2:**
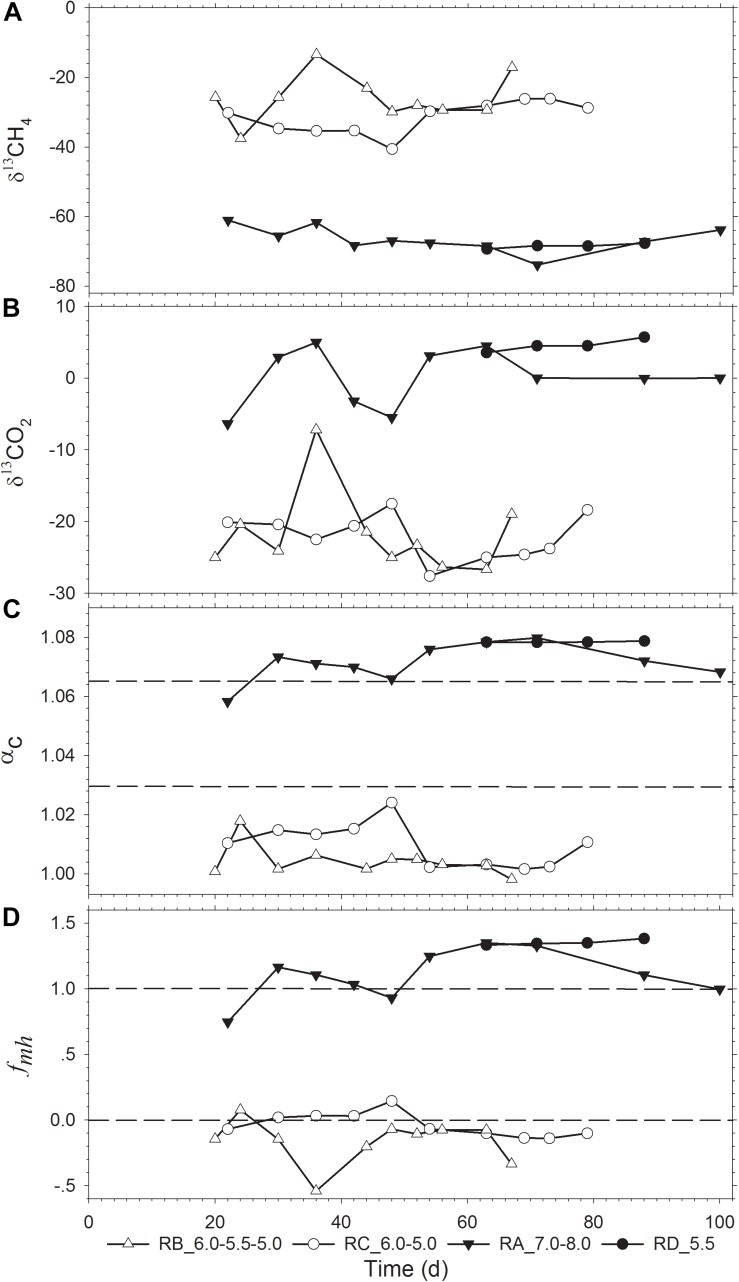
Temporal change of the stable isotopic indicators **(A)** δ^13^CH_4_ and **(B)** δ^13^CO_2_, and evaluation of **(C)** α_c_ and **(D)** *f*_mh_. δ^13^CH_4_ and δ^13^CO_2_ are the ^13^C isotope signatures of the produced CH_4_ and CO_2_; *f*_mh_ is the fraction of CH_4_ produced through the hydrogenotrophic pathway (HM); α_c_ is the apparent fractionation factor.

The isotope values and temporal changes of δ^13^CH_4_, δ^13^CO_2_, apparent fractionation factor (α_c_), and *f*_mh_ (fraction of CH_4_ produced through HM) are shown in Figure 2. The isotopic data were absent for the RE_5.0 reactor as no gas was produced in this reactor at pH 5.0. The four reactors could be divided into two groups according to the range of δ^13^CH_4_ values. The RA_7.0-8.0 and RD_5.5 reactors were grouped together with the δ^13^CH_4_ ranging from −75‰ to −60‰, while the RB_6.0-5.5-5.0 and RC_6.0-5.0 reactor groups had δ^13^CH_4_ ranging from −40‰ to −10‰. Additionally, pH adjustment had a slight influence on the value of δ^13^CH_4_. These results could also be attributed to the change in δ^13^CO_2_, which coincided with δ^13^CH_4_ fluctuation (Figures 2A,B).

The α_c_ and *f*_mh_ values were calculated to evaluate the contribution of different methanogenic pathways. The four reactors could also be divided into the same two groups according to the α_c_ and *f*_mh_ values (Figures 2C,D). The α_c_ values of the RA_7.0-8.0 and RD_5.5 reactors ranged from 1.058 to 1.080, which indicated that HM was the predominant methane production pathway. Contrastingly, the α_c_ value of the RB_6.0-5.5-5.0 and RC_6.0-5.0 reactors ranged from 1.000 to 1.024, which indicated that methane was mainly produced via the acetoclastic pathway. Accordingly, the calculated *f*_mh_ values revealed a similar conclusion (Figure 2D). It should be noted that *f*_mh_ is a parameter that is a simplified composite of α_m__h_, α_m__*a*_, δ^13^CH_3_COO^–^, and δ^13^CO_2_ (Conrad, 2005). Thus, there were some sample points with the *f*_mh_ value of >1.0 or <0 in our calculation.

Generally, low pH environment is not considered to be favorable for methanogenesis. Additionally, the gas production rate could be greatly limited with the HM pathway becoming the predominant pathway under acidic shock (Hao et al., 2012). However, we demonstrated that AM was still the dominant pathway in the acidic environment by gradually decreasing pH (RB_6.0-5.5-5.0, RC_6.0-5.0), which shifted to HM pathway when the pH was directly decreased to a low value (RD_5.5).

### Microbial Community Structure With Different pH Regulating Strategies

**FIGURE 3:**
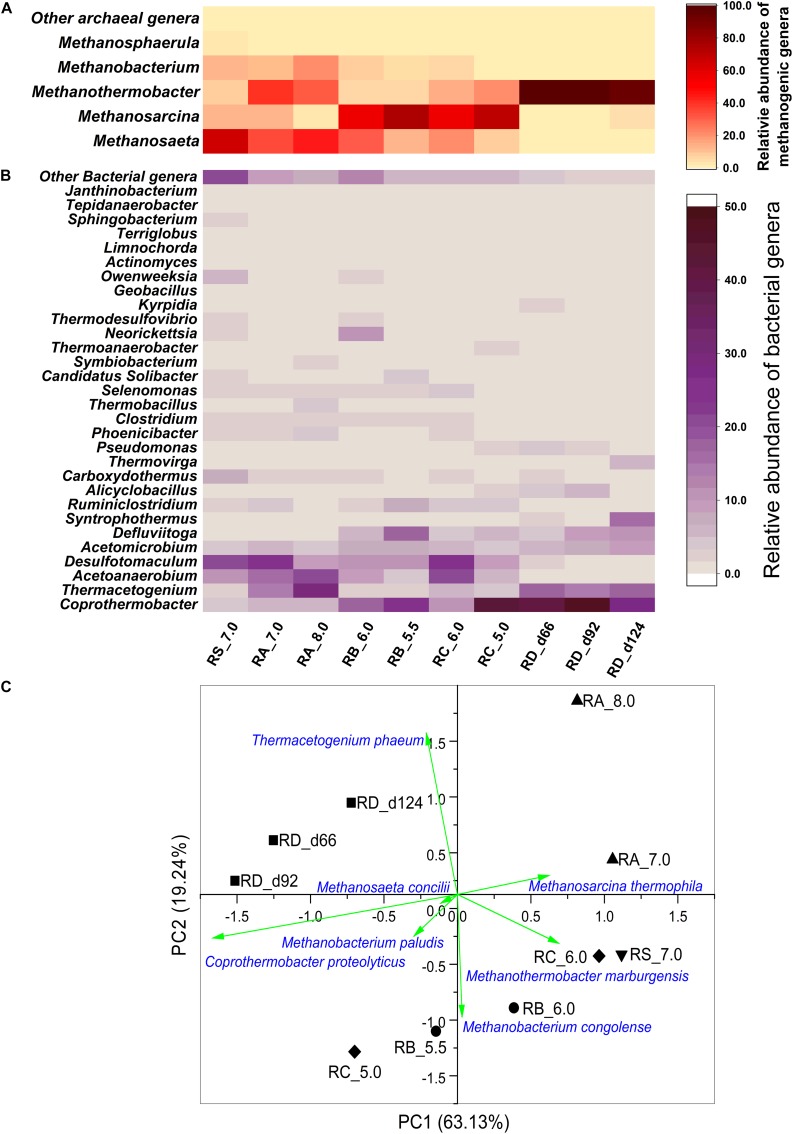
Heatmap of **(A)** archaeal and **(B)** bacterial composition of the microbial communities at the genus level in different reactors based on the 16S rRNA data. Only genera with relative percentage higher than 1% are shown. The genera with relative percentage lower than 1% were aggregated as Other archaeal genera and Other bacterial genera. **(C)** Principal component analysis (PCA) based on the relative abundance of microbial species. (▼) Reactor RS_7.0, (▲) Reactor RA_7.0-8.0, (●) Reactor RB_6.0-5.5-5.0, (◆) Reactor RC_6.0-5.0, (■) Reactor RD_5.5.

The microbial community structure of the anaerobic reactors at the genus level under various pH values is shown in Figures 3A,B. Abundant genera were evident in all the reactor consortia. The bacterial community mainly comprised *Coprothermobacter*, *Thermacetogenium*, *Acetoanaerobium*, *Desulfotomaculum*, and *Acetomicrobium*. Among the archaeal community, the methanogens comprised the predominant fraction. When the pH values of the reactors varied, the microbial community structure changed with a decrease in both microbial richness and diversity (Supplementary Table S2).

Among the archaea, the relative abundance of *Methanosarcina* increased when the pH was decreased gradually in the RB_6.0-5.5-5.0 and RC_6.0-5.0 reactors. *Methanosarcina*, a methanogenic genus capable of both AM and HM, was reported to play an important role in initiating methanogenesis from the double stress (pH 5.0–6.5 and high acidity) (Hori et al., 2006; Staley et al., 2011), while the methanogenic pathway of this genus was not clear. Although *Methanosarcina* was suggested for heavy-duty biomethanation with a pH shock of 0.8–1.0 units and acetate concentration up to 250 mM, the hydrogenotrophic pathway was reported to be the dominant methanogenic pathway (De Vrieze et al., 2012). The hydrogenotrophic pathway was reported to be the predominant pathway in several digesters under acid crisis (Hao et al., 2012). However, based on the isotopic information in Figure 2, *Methanosarcina* in the acidic reactors (RB_6.0-5.5-5.0 and RC_6.0-5.0) was suggested to promote the AM pathway. However, *Methanothermobacter* was the dominant microbe in the RD_5.5 reactor when the pH was adjusted to 5.5 from 7.0 directly. *Methanosaeta*, which was prevalent in the inoculum, decreased in all the studied consortia when the pH was changed regardless of an increase or decrease in pH or a sudden or gradual decrease in pH.

Among the bacteria, the relative abundance of *Coprothermobacter* increased markedly in all the reactors when the pH was decreased. The abundance of *Thermacetogenium* increased when the pH was decreased directly to 5.5 (RD_5.5) or increased to 8.0 (RA_7.0-8.0) and decreased with the gradual decline in pH (RB_6.0-5.5-5.0 and RC_6.0-5.0). *Thermacetogenium* is a known SAOB that can produce H_2_ and CO_2_ from acetate (Hattori et al., 2000, 2005)_ENREF_23. The abundance of *Acetoanaerobium* and *Desulfotomaculum* decreased when the pH was reduced gradually but did not markedly change when the pH was increased or reduced suddenly. There was also no large diversification in the relative abundance of *Acetomicrobium* before and after the pH was changed. The relative abundance of *Defluviitoga* increased when the pH was reduced directly or gradually, while no obvious change occurred when the pH was increased compared to that in the inoculum.

Principal component analysis based on the Bray–Curtis distances was conducted using the relative abundance of microbial members in the mixed culture. As shown in Figure 3C, the samples were clustered together with various pH adjustment strategies. This indicated that the microbial communities gradually adapted to different pH environments. The RA_8.0 sample was distant from all other samples and was an indicator that the microbial community structure in the alkaline environment was different from that of the inoculum and other cultures in the acidic environment. The RB_6.0-5.5-5.0 and RC_6.0-5.0 cultures were clustered distinctly from the RD_5.5 culture, although they were all operated under the acidic environment. This indicated that the microbial community structures varied in response to various patterns of pH adjustment strategies.

### Comparative Metagenomic and Metatranscriptomic Analyses of the Consortium That Adapted to an Extremely Acidic Environment

To gain in-depth knowledge of the methanogenic consortium that can still produce methane under extremely acidic conditions with a high acetate concentration and low pH in the RD_5.5 (free acetic acid concentration up to 15.84 mM) reactor, we conducted a comparative analysis of the metagenome and metatranscriptome.

**FIGURE 4:**
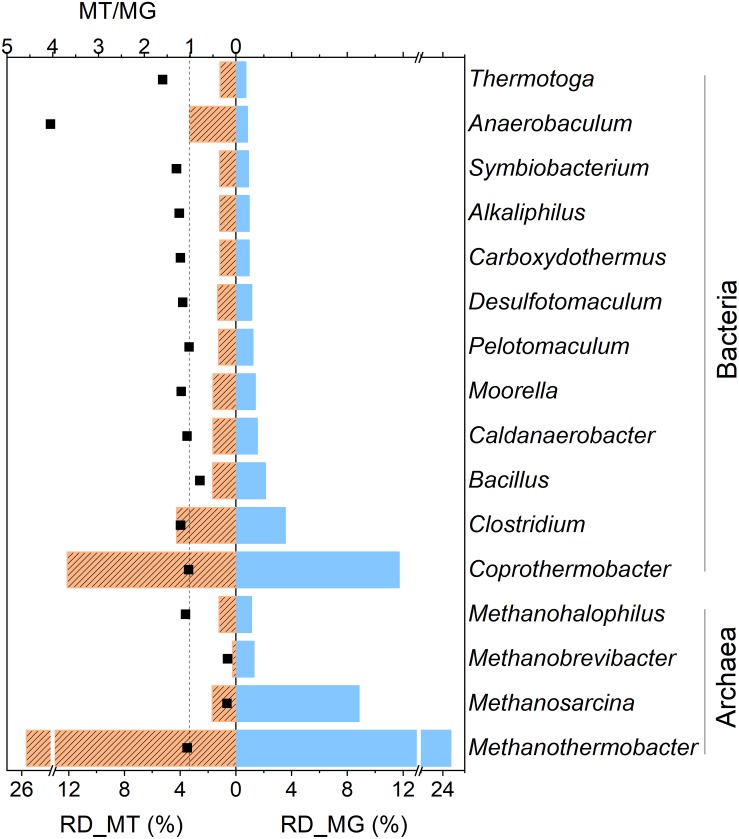
Relative abundance levels of the dominant genera of the metagenome (RD_MG, blue column) and metatranscriptome (RD_MT, orange column) of the RD_5.5 reactor. Only the relative abundance values of the identified genera higher than 1% in DNA or the cDNA library are listed. The black dots (MT/MG) indicate the ratio of the relative abundance values of those taxa from the metatranscriptome and metagenome. The dashed line indicates that the value of MT/MG is 1.

Taxonomic annotation revealed the high microbial richness and diversity of the studied methanogenic consortium derived from both the metagenomic and metatranscriptomic datasets(Supplementary Table S2). The details of the taxonomic composition at various levels are shown in Supplementary Table S3. *Methanothermobacter* and *Coprothermobacter* were the predominant archaeal and bacterial members, respectively. *Methanosarcina* and *Clostridium* were also abundant. At the species level, the *Methanothermobacter* genus was mainly comprised *M. thermautotrophicus* and *M. marburgensis*, while the *Coprothermobacter* genus only included *C. proteolyticus*. *M. barkeri* and *M. mazei* were the predominant species of the *Methanosarcina* genus. *Clostridium*, one of the abundant bacterial genera, comprised various species, such as *C*. *thermocellum*, *C*. *botulinum*, *C*. *difficile*, *C*. *perfringens*, *C. cellulolyticum*, and *C. acetobutylicum*. The relative abundance of these microorganisms was similar in the metagenome and metatranscriptome with each other. This indicated that these highly abundant microbes also exhibited high metabolic activities, except for a few members (*Anaerobaculum*, *Methanosarcina*, and *Methano brevibacter*). The MT/MG ratio was defined as the ratio of the relative abundance value of a taxon in the metatranscriptome to compared that in the metagenome, which was usually used to roughly describe the *in situ* activities of microbial members in the mixed microbial communities. As shown in Figure 4, the *Anaerobaculum* genus had a low genomic abundance (0.82%) but exhibited a MT/MG ratio of 4.02. This indicated that the members of this genus had high relative transcriptomic abundance (3.30%), genes of which were highly expressed. Contrarily, the *Methanosarcina* genus contributed to 8.83% of the metagenome and had a MT/MG ratio of 0.19. This indicated that the genes of this genus were transcribed at a much lower level. In this study, most of the *Methanosarcina* members were probably inhibited or even killed by the low pH environment, but the genes of dead cells could still be detected by DNA-based methods. A similar phenomenon was observed for *Methano brevibacter* (MT/MG of 0.18). Nevertheless, the MT/MG values of other important microbes, such as *Methanothermobacter* and *Coprothermobacter* were all approximately 1 (Figure 4), which indicated that these microbes were both abundant and actively participated in the methanation process.

Global analysis of the genes in the metagenome and metatranscriptome was conducted to understand the acid adaptation mechanism of the microbiota. The relative abundances of 5,237 genes and corresponding transcripts annotated with the Subsystem as the reference database were quantified. The overall gene relative abundance values and corresponding transcript relative abundance values were generally correlated (Pearson’s coefficient; *r* = 0.825). However, the transcript abundance values of several gene families were an order-of-magnitude higher or lower than expected from their DNA abundance (indicating genes overexpressed or under-expressed). The log MT/MG ratios of genes belonging to specific subjects of gene families were computed to identify differently regulated transcripts.

**FIGURE 5:**
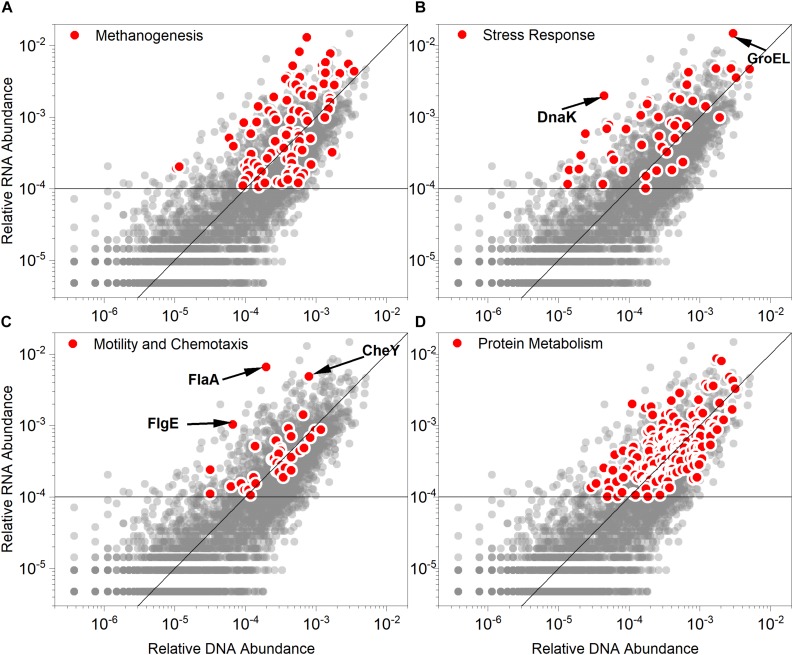
Relative abundance values of the gene (DNA) and corresponding transcript (RNA) in the **(A)** methanogenesis, **(B)** stress response, **(C)** motility and chemotaxis and **(D)** protein metabolism modules (red points). Each scatterplot illustrates the gene and transcript relative abundance based on Subsystem databases (gray points). The function line of *y* = *x* was graphed to distinguish between the overexpressed (points above the line, indicating RNA > DNA) and under-expressed genes (points below the line, indicating RNA < DNA). The horizontal line indicates that the relative RNA abundance was 0.01%. GroEL, heat shock protein family chaperone; DnaK chaperone protein; CheY, chemotaxis regulator and transmits chemoreceptor signals to flagellar motor component; FlaA, flagellin protein; FlgE, flagellar hook protein.

The genes regulating methanogenesis, stress response, motility and chemotaxis, and protein metabolism were among the strongly overexpressed genes (Figure 5). The genes were also assigned to specific species to estimate their contributions to the community (Supplementary Tables S4, S5). Although some genes contributing to methanogenesis were under-expressed, most genes regulating methanogenesis were actively transcribed with high abundance (>0.01%). This explains the decreased but non-stop methane production activities. Generally, the genes of the stress response cluster were overexpressed. The genes of this cluster were usually reported to play an important role in the microbial acid tolerance mechanism (Heyde and Portalier, 1990; Foster, 1993; Hartke et al., 1996). Several stress responses are known to interact with pH stress and pH resistance, including oxidative stress, heat shock, and envelope stress (Maurer et al., 2005). Apart from some general stress response genes, typical genes encoding shock responding proteins, such as GroEL and DnaK (Karem et al., 1994) were pronouncedly overexpressed. In this study, GroEL, mainly derived from *Anaerobaculum*, *Coprothermobacter*, and *Thermacetogenium* (Supplementary Table S4), had a high MT/MG ratio (4.98) and extremely high transcriptional abundance (1.49%). The MT/MG ratio was also as high as 1.73 for DnaK, which indicated that the overexpression of this gene was vital for the acid resistance of the consortium. Additionally, *Anaerobaculum mobile* and *Coprothermobacter proteolyticus* contributed the most to the abundance of DnaK gene and the corresponding transcript in the community (Supplementary Table S4). Some genes encoding proteins required for sporulation of this cluster were actively transcribed, such as SpoVS (MT/MG = 24.51) and SpoVG (MT/MG = 7.49). This indicated that some microbes respond to acid stress by sporulation. Additionally, the genes in the motility and chemotaxis cluster were also highly transcriptionally active, especially FlaA (MT/MG = 33.25), which encodes flagellin. The gene family involved in archaeal flagellar biosynthesis and function was weakly overexpressed and comprised genes, such as FlaB (MT/MG = 7.56), Flgl (MT/MG = 3.72), FlgG (MT/MG = 2.25), FlgB (MT/MG = 2.22), FliF (MT/MG = 2.22), and FlgE (MT/MG = 2.14). Acetate has been reported to induce the flagellar regulon and enhance motility (Polen et al., 2003). Additionally, the expression of flagellar synthesis genes was strongly stimulated at low pH (Maurer et al., 2005). Only a few methanogens were reported to have flagella, which mainly belong to the *Methanococcus* genus, such as *Methanococcus voltae*, *Methanococcus maripaludis*, *Methanococcus thermolithotrophicus*, and *Methanococcus jannaschii* (Thomas and Jarrell, 2001). However, *Methanococcus voltae*, the species in which flagellation of archaea has been most frequently deeply studied, is mesophilic (Bayley and Jarrell, 1999) and was not detected in our studied system. The predominant methanogens in this study, such as *Methanothermobacter*, *Methanosaeta*, and *Methanosarcina* had no flagella. The sources of the highly transcribed flagellum-related genes were *Thermacetogenium phaeum* and *Coprothermobacter proteolyticus* (Supplementary Table S5). The CheY gene, which encodes a chemotaxis regulator, was transcriptionally regulated by pH (Maurer et al., 2005). The CheY gene was highly expressed (MT/MG = 6.03) along with the flagellin-related genes. *Coprothermobacter proteolyticus* also made valuable contributions to the overexpression of CheY. The abundant protein metabolism-related genes were also highly expressed, which can be explained by the predominance of *Coprothermobacter proteolyticus*.

**FIGURE 6:**
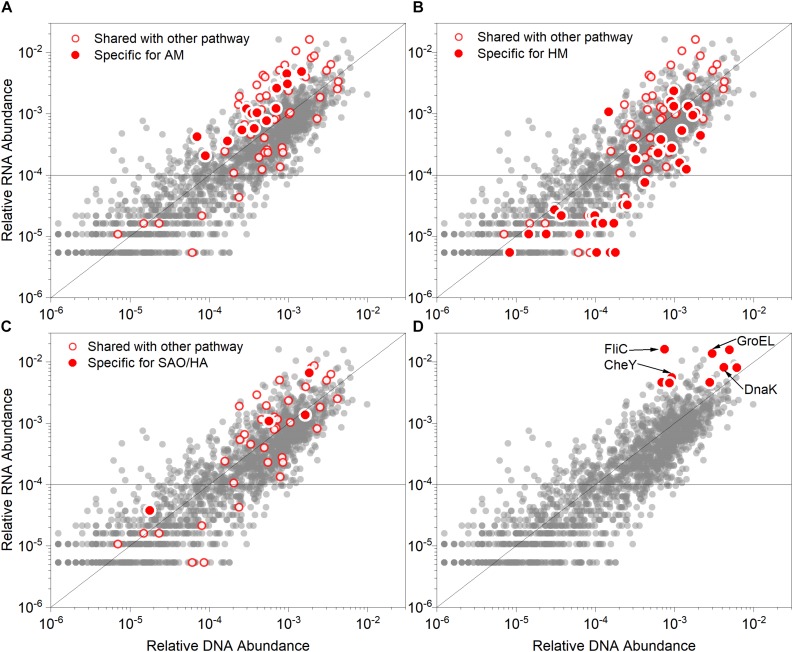
Relative abundance values of genes (DNA) and corresponding transcripts (RNA) associated with the metabolisms of **(A)** acetoclastic methanogenesis (AM), **(B)** hydrogenotrophic methanogenesis (HM), **(C)** Syntrophic acetate oxidation/Homoacetogenesis (SAO/HA) and **(D)** prominent overexpressed genes. The red points in **(A–C)** indicate genes specific for the described pathways, while the red circles represent genes shared with other methanogenic pathways. The red points in **(D)** highlight the prominent genes (both highly overexpressed and abundant). Each scatterplot illustrates the gene and transcript relative abundance based on KO databases (gray points). The function line of *y* = *x* was graphed to distinguish between overexpressed (points above the line, indicating RNA > DNA) and under-expressed genes (points below the line, indicating RNA < DNA). The horizontal line indicates that the relative RNA abundance was 0.01%. GroEL, chaperonin GroEL; DnaK, molecular chaperone; CheY, chemotaxis family, response regulator; FliC, flagellin.

To identify the specific active pathway of acetate methanation and acid stress response genes in this study, the total reads of the metagenome and metatranscriptome were annotated based on KEGG and KO databases. In total, 2,061 KOs were identified and the relative abundance values of DNA correlated with that of RNA (Pearson’s coefficient; *r* = 0.876). We focused on the genes encoding the enzymes participating in various methanation pathways (SAO-HM and AM) (Figures 6A–C), which are listed in Supplementary Table S6. The detailed information on the enzymes and corresponding subunits is shown in Supplementary Table S7. The value of the MT/MG ratio ranged from 0.10 to 8.36 (Supplementary Table S6), which indicated that several genes encoding specific methanogenic enzymes were actively transcribed and others were inhibited. As shown in Figures 6A–C, the genes encoding enzymes involved in the HM, AM, and SAO/HA pathways exhibited a wide range of abundance and MT/MG ratios. However, the relative abundance of RNA specific for AM was more than 0.01% with MT/MG values more than 1. The genes for HM did not appear to be actively transcribed. However, the isotopic analysis indicated that methane was mainly produced from HM. This may be because of the overall high abundance of SAO-HM pathway-related microbes and genes, although the AM-related genes were highly expressed. The genes of the stress response and motility and chemotaxis clusters were confirmed to be highly overexpressed, especially GroEL, DnaK, CheY (Figure 6D), and flagellum-related genes (FliC), which are mainly derived from *Anaerobaculum*, *Thermacetogenium phaeum*, and *Coprothermobacter proteolyticus* (Supplementary Tables S4, S5).

## Discussion

### Microbial Community Structure Reconstruction and Methanogenesis Pathway Shift in Response to Sudden and Gradual Acidification

The microbial community structures varied with the pH. However, sudden acidification shock or gradual acclimation to low pH markedly affected the microbial community structuredifference.

**FIGURE 7:**
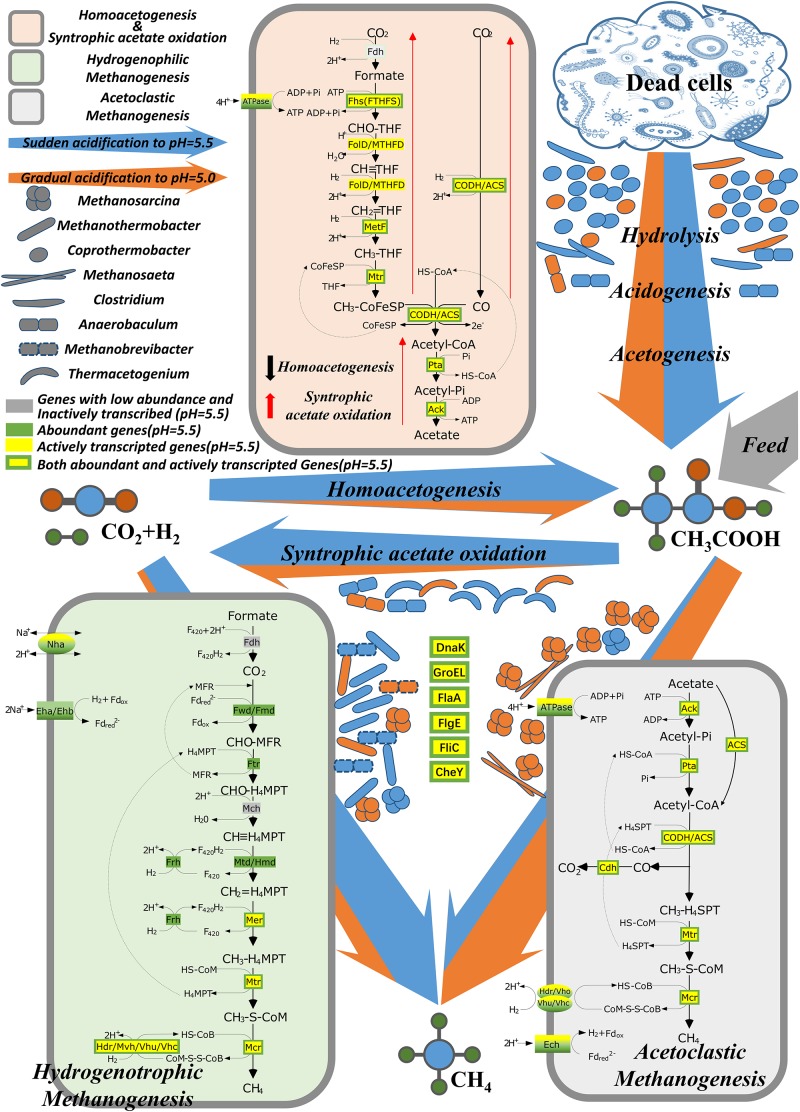
Microbial networks for thermophilic methanogenesis under an extremely acidic environment. Blue arrow, dominant pathway under sudden acidification to pH 5.5; orange arrow, dominant pathway under gradual acidification to pH 5.0.

The high acidification levels in the RD_5.5 and RE_5.0 reactors resulted in the immediate inhibition of gas production in methanogenic systems. Nevertheless, 72.6% of the initial methane production rate recovered after a 42-day lag phase only for the RD_5.5 reactor but not for the other reactors at pH 5.0. This indicated that sudden acidification to pH ≤ 5.0 is lethal to the thermophilic AD systems with high organic loading. However, the system could survive the acidification stress at pH ≥ 5.5. The mechanism underlying the survival of microbes and methane production is interesting. The results of this study indicated that most of the methanogens in the inoculum, such as *Methanosarcina*, *Methanosaeta*, and *Methanobacterium*, as well as other acid-sensitive bacteria, were inhibited/killed by sudden acidification. During the long lag phase, the acid-tolerant scavengers (mainly *Coprothermobacter*) decomposed the dead cells and other organic molecules (protein and saccharides) in the culture medium to acetate and other compounds (amino acids and pyruvic acid). Acetate-oxidizing bacteria (mainly *Thermacetogenium*) converted acetate into H_2_ and CO_2_, both from the feed organics and from hydrolysis, which was then utilized syntrophically by the hydrogenotrophic methanogens (mainly *Methanothermobacter*) to produce methane (Figure 7). Notably, the predominant genus in the thermophilic anaerobic digester was reported to be *Coprothermobacter* (Gagliano et al., 2015), which may also function as SAO (Lu et al., 2014; Kunath et al., 2019). The high α_c_ and *f*_mh_ values supported the role of HM pathway in methane production.

The microbial composition changed when the pH was decreased gradually. The acclimation process increased the acid resistance of the consortia irrespective of the pH decrease (from 7.0 to 5.0) in 3 stages (RB_6.0-5.5-5.0) or 2 stages (RC_6.0-5.0). Both systems still worked for a short period at pH 5.0. Although a short lag phase (4 and 6 days for RB and RC, respectively), was observed, the gas production rates decreased markedly and finally decreased to 0 (no gas production). Thus, the extremely acidic environment (pH < 5.0) was inhibitory for such thermophilic AD systems, although the acclimation process was feasible from the perspective of long-term operation. However, compared to the microbial communities in the sudden acidification groups, the microbial communities in the gradual acidification groups exhibited variable responses. After pH adjustment, stable isotopic indicator analysis indicated that approximately all the methane was produced by AM, which coincided with the abundance of the acetoclastic methanogen, *Methanosarcina* and the rare hydrogenotrophic methanogen, *Methanothermobacter* in the culture. The gas production rates even exceeded those of the initial inoculum system.

When the pH was changed from neutralto slightly alkaline (pH 8.0), there was no marked change in the microbial community structure. However, the microbial richness increased and diversity decreased slightly with a concomitant decrease in the methane production rate. This indicated that the alkaline environment inhibited the predominant microbial members in the original inoculum and acted as a selection pressure for few microbes surviving from the acid stress, such as *Methanothermobacter* and *Thermacetogenium*. The prevalence of *Methanothermobacter* and *Thermacetogenium* in the culture may also provide an explanation for the dominant SAO-HM pathway derived from the stable isotopic signature analysis.

### Microbial Consortium Self-Adaptation to an Extremely Acidic Environment

It is interesting to analyze the survival of consortium under extreme acidic environment and regained the methane-producing ability after the lag phase. The genes in the metagenome and metatranscriptome were analyzed to elucidate the complex consortium structure, metabolic characteristics, and transcriptional regulation under such a tough environment. The consortium predominantly comprised *Methanothermobacter* and *Coprothermobacter*, which were both in high abundance and actively participated in the metabolism. The metatranscriptome analysis revealed that *Methanosarcina* exhibited low abundance. This indicated that most of the *Methanosarcina* genus was inhibited in the sudden and long-term acidification at pH 5.5. *Anaerobaculum*, another low abundant microbe, was transcriptionally active and could ferment peptides and saccharide metabolites along with *Coprothermobacter* (Menes and Muxi, 2002; Maune and Tanner, 2012). Methane was produced from the HM pathway at a low production rate. The genes encoding the HM-related enzymes were generally in higher abundance and actively transcribed. which indicated the dominance of the HM pathway. These data demonstrated that *Methanothermobacter* was the robust methane producer via the SAO-HM pathway under an extremely acidic environment with high organic loading. This microbe utilized acetate both from feeding and scavengers or fermenters (mainly *Coprothermobacter*, *Clostridium*, and *Anaerobaculum*).

Numerous studies have investigated the adaptive response to fatty acids or the acid-tolerant mechanism of eukaryotic organisms, such as *Saccharomyces cerevisiae* and *Zygosaccharomyces bailii (Piper et al., 2001)* and prokaryotic microbes, such as *Lactobacillus sanfranciscensis (De Angelis et al., 2001)*, *Lactococcus lactis* subsp. *Lactis (Hartke et al., 1996)*, *Salmonella typhimurium (Foster and Hall, 1990)*, and *Escherichia coli* (Polen et al., 2003). However, the limitation of this study is that there are limited data on the genetic response of methanogenic archaea to acidification and extreme acidification (both high loading rates and low pH, resulting in a high level of free acidic molecules). Nevertheless, the results of this study indicated that the acid-tolerant mechanism and gene regulation patterns in response to acidification were similar between the studied consortium and pure cultures. The genes encoding proteins of the general stress response and chemotaxis and flagellum, especially GroEL, DnaK, FlaA, FlgE, FliC, and CheY were highly expressed in response to acidification. The overexpression of these genes was also observed in the culture repressed with long-chain fatty acids (oleate) (Treu et al., 2016). Additionally, the microbial communities reassembled its community structure and established syntrophism between SAOB and hydrogenotrophic methanogens to release the acidic stress from feeding acetate and that produced from the acidogenic bacteria.

## Conclusion

This study simultaneously investigated the reactor performances, taxonomic composition, and the genes associated with methanogenesis using metagenomic and metatranscriptomic approaches under various patterns of acidification. The main findings are:

(1) The results confirmed that the long-term survival of an acid-tolerant methanogenic consortium (in the form of flocs, but not granules) in an artificial anaerobic digester at pH 5.5 was feasible. Syntrophy was established between *Coprothermobacter* and hydrogenotrophic methanogens to resist system acidification.
(2) *Methanosarcina* can be markedly inhibited by sudden acidification to pH 5.5. However, it can survive the stepwise decrease in pH to 5.0.
(3) The predominant methanogen was *Methanosarcina*, which produced methane via AM when the pH was gradually decreased. Contrastingly, the consortia self-adapted to sudden acidification by increasing the abundance of *Methanothermobacter* and methane was produced by the tandem pathway of SAO and HM.
(4) The source of genes of the stress response, motility and chemotaxis clusters was *Anaerobaculum*, *Thermacetogenium phaeum*, and *Coprothermobacter proteolyticus*. The abundance of these genes in the metatranscriptome was higher than that in the metagenome, which indicated that these genes were actively expressed and probably were functional under the strong acidic conditions.
(5) *Coprothermobacter* is vital for the methanogenic consortium to resist acidification and recover methane production in extremely acidified thermophilic anaerobic digesters.

The findings of this study elucidated the acid-resistant mechanism of thermophilic methanogenic consortium and improved our understanding of the function of *Methanosarcina*, *Methanothermobacter*, and *Coprothermobacter* and the microbial characteristics under extreme acidic environment, which could be utilized to design more effective and robust thermophilic anaerobic digester for high organic loading wastewater or waste treatment.

## Data Availability Statement

All the 16S rRNA sequences were uploaded to the National Center for Biotechnology Information (NCBI) database (accession number: SUB5012618). The raw metagenomic and metatranscriptomic sequences are available on MG-RAST (http://metagenomics.anl.gov) under the accession numbers, mgm4798897.3 and mgm4798898.3, respectively. Other datasets used and/or analyzed during this study are available from the corresponding author on reasonable request.

## Author Contributions

WH analyzed the data and drafted the manuscript. YL conducted the experiment. PH, LS, and FL developed the general research question, discussed the results, and revised the manuscript. All authors approved the final version of the manuscript.

## Conflict of Interest

The authors declare that the research was conducted in the absence of any commercial or financial relationships that could be construed as a potential conflict of interest.

## Footnotes

1 http://metagenomics.anl.gov

## Abbreviations

AD: anaerobic digestion
AM: acetoclastic methanogenesis
HA: homoacetogenesis
HM: hydrogenotrophic methanogenesis
SAO: syntrophic acetate oxidation
SAOB: syntrophic acetate-oxidizing bacterium
VFAs: volatile fatty acids.
