# Supplementary material for: A Methanogenic Consortium Was Active and Exhibited Long-Term Survival in an Extremely Acidified Thermophilic Bioreactor
Source: Front Microbiol. 2019 Nov 26;10:2757. doi: 10.3389/fmicb.2019.02757 (PMC6988822; doi:10.3389/fmicb.2019.02757)
Supplement: Supplementary file 1 [file Data_Sheet_1.docx]

Supplementary Material

**Text: Section 1;**

**Tables: Table S1-S4 and S6 (Table S5 and S7 are provided as individual .xlsx file).**

# Section 1: Set up of an anaerobic reactor for cultivating seed inoculum.

Methanogenic sludge flocs were cultivated in a 16-L anaerobic sequenced batch reactor (ASBR; marked as RS7.0) holding 12 L medium, which was stably run for two months under a neutral environment (pH = 7.0). The temperature of the reactor was kept at 55 °C by water bath. Each cycle of reactor operation included substrate filling for 0.5 hours, anaerobic reaction in the batch mode for 45 hours, sludge flocs settlement for 2 hours, and effluent discharge for 0.5 hours, that was two days per cycle. The ASBR was filled with 1.2 L of fresh medium with acetate as the sole organic carbon source every cycle of operation, and the initial acetate concentration inside the reactor was kept at 104 mM. Next, the pH was adjusted back to 7.0 with 3 M of NaOH. After the filling period, the influent supply was stopped. After 45 hours of reaction, the stirrer was stopped to allow settlement of the sludge flocs. In the remaining 30 min of the total cycle, 1.2 L of the supernatant was discharged with N_2_ purging to prevent the entry of air. The fresh medium was concentrated acetate solution dissolved in nutrients buffer, which contained per liter 1.0 g NH_4_Cl, 0.4 g K_2_HPO_4_·3H_2_O, 0.2 g MgCl_2_·6H_2_O, 0.08 g CaCl_2_·2H_2_O, 0.2 g Na_2_S·9H_2_O, 10 mL trace element solution and 10 mL stock vitamin solution. The trace element solution contained per liter: 400 mg FeCl_2_·4H_2_O, 19 mg H_3_BO_3_, 100 mg ZnCl_2_, 20 mg CuCl_2_·2H_2_O, 100 mg MnCl_2_·4H_2_O, 10 mg Na_2_MoO_4_·4H_2_O, 90 mg AlCl_3_·6H_2_O, 170 mg CoCl_2_.6H_2_O, 20 mg NiCl_2_·6H_2_O, 194 mg Na_2_SeO_3_·5H_2_O, 1.0 g EDTA-2Na. The stock vitamin solution contained per liter: 10 mg biotin, 50 mg pyridoxine hydrochloride, 25 mg thiamine hydrochloride, 25 mg D-calcium pantothenate, 10 mg floic acid, 25 mg riboflavin, 25 mg nicotinic acid, 25 mg P-aminobenzic acid and 0.5 mg vitamin B_12_.

# Table S1. Average methane production rates during different operational periods under various pH conditions.

| Reactor | RS_7.0 | RA_7.0-8.0 | | RB_6.0-5.5-5.0 | | | RC_6.0-5.0 | | RD_5.5 | RE_5.0 |
| --- | --- | --- | --- | --- | --- | --- | --- | --- | --- | --- |
| pH | 7.0 | 7.0 | 8.0 | 6.0 | 5.5 | 5.0 | 6.0 | 5.0 | 5.5 | 5.0 |
| Period (Day) | 0˗14 | 14˗56 | 56˗100 | 14˗36 | 36˗56 | 56˗100 | 14˗54 | 54˗100 | 14˗100 | 14˗100 |
| Methane production rate (mL/d) | 260.3 | 399.4 | 79.5 | 778.7 | 618.1 | 98.3 | 813.9 | 161.3 | 87.3 | 0.0 |
| Period (Day)* | / | / | / | / | 42-56 | 60-67 | / | 60-73 | 56-88 | / |
| Methane production rate (mL/d) * | / | / | / | / | 877.0 | 570.0 | / | 495.1 | 189.0 | / |
| Feed rate (acetate)  (mmol/d) | 12.61 | 21.83 | 9.12 | 36.74 | 44.33 | 27.81 | 38.33 | 29.46 | 13.97 | / |

Note: * The gas production periods excluded the lag phase period without or negligible methane production.

# Table S2. Microbial community richness and diversity for each reactor consortia.

| Reactor | Consortium | OTUs | | | ACE^1^ | Shannon Index^2^ |
| --- | --- | --- | --- | --- | --- | --- |
|  |  | Bacteria | Archaea | All |  |  |
| RS_7.0 | RS_7.0 | 227 | 16 | 243 | 33,782 | 3.44 |
| RA_7.0-8.0 | RA_7.0 | 172 | 11 | 183 | 37,096 | 2.96 |
|  | RA_8.0 | 313 | 15 | 328 | 39,686 | 2.74 |
| RB_6.0-5.5-5.0 | RB_6.0 | 217 | 12 | 229 | 36,251 | 3.21 |
|  | RB_5.5 | 166 | 8 | 175 | 19,242 | 2.8 |
| RC_6.0-5.0 | RC_6.0 | 130 | 11 | 142 | 58,517 | 2.76 |
|  | RC_5.0 | 341 | 13 | 354 | 38,783 | 2.5 |
| RD_5.5 | RD_d66 | 246 | 10 | 256 | 42,781 | 2.2 |
|  | RD_d92 | 182 | 9 | 191 | 29,240 | 1.94 |
|  | RD_d124 | 225 | 8 | 233 | 53,217 | 2.23 |
|  | RD_MG | / | / | / | 915,532 | 1.82 |
|  | RD_MT | / | / | / | 416,535 | 1.81 |

Notes: ^1^ ACE richness estimator: the total number of OTUs estimated by infinite sampling. A higher number indicates higher richness;

^2^ Shannon’s diversity index: an index to characterize species diversity. A higher value indicates more diversity.

# Table S3. Taxonomic affiliation at different levels based on DNA and cDNA sequences from RefSeq database.

| Taxonomic Level | Microbes | Relative abundance  of MG (%) | Relative abundance  of MT (%) |
| --- | --- | --- | --- |
| Domain | Bacteria | 57.41 | 66.05 |
|  | Archaea | 42.28 | 33.67 |
|  | Eukaryota | 0.27 | 0.26 |
|  | Unclassified | 0.04 | 0.02 |
| Phylum | Euryarchaeota | 41.94 | 33.34 |
|  | Firmicutes | 40.42 | 43.59 |
|  | Proteobacteria | 6.23 | 6.09 |
|  | Thermotogae | 1.78 | 3.02 |
|  | Synergistetes | 1.51 | 5.55 |
|  | Actinobacteria | 1.38 | 1.64 |
|  | Chloroflexi | 1.22 | 1.05 |
|  | Cyanobacteria | 0.86 | 0.65 |
|  | Bacteroidetes | 0.67 | 0.66 |
|  | Deinococcus-Thermus | 0.50 | 0.56 |
|  | Aquificae | 0.39 | 0.40 |
|  | Nitrospirae | 0.36 | 0.25 |
|  | Spirochaetes | 0.33 | 0.43 |
|  | Dictyoglomi | 0.30 | 0.31 |
|  | Crenarchaeota | 0.28 | 0.29 |
|  | Fusobacteria | 0.25 | 0.37 |
|  | Chlorobi | 0.24 | 0.24 |
|  | Acidobacteria  Planctomycetes | 0.24 | 0.29 |
|  |  | 0.17 | 0.21 |
|  | Unclassified (derived from Bacteria) | 0.15 | 0.20 |
| Class | Clostridia | 33.63 | 37.20 |
|  | Methanomicrobia | 27.89 | 29.09 |
|  | Methanobacteria | 11.40 | 2.21 |
|  | Bacilli | 5.79 | 5.13 |
|  | Deltaproteobacteria | 2.62 | 2.66 |
|  | Thermotogae (class) | 1.78 | 3.02 |
|  | Gammaproteobacteria | 1.52 | 1.42 |
|  | Synergistia | 1.51 | 5.55 |
|  | Actinobacteria (class) | 1.38 | 1.64 |
|  | Alphaproteobacteria | 1.08 | 1.07 |
|  | Methanococci | 1.03 | 0.45 |
|  | Negativicutes | 0.84 | 0.97 |
|  | Betaproteobacteria | 0.80 | 0.71 |
|  | Unclassified (derived from Cyanobacteria) | 0.79 | 0.61 |
|  | Thermococci | 0.67 | 0.72 |
|  | Chloroflexi (class) | 0.62 | 0.52 |
|  | Deinococci | 0.50 | 0.56 |
|  | Archaeoglobi | 0.45 | 0.42 |
|  | Aquificae (class) | 0.39 | 0.40 |
|  | Nitrospira (class) | 0.36 | 0.25 |
| Order | Methanobacteriales | 27.13 | 28.60 |
|  | Thermoanaerobacterales | 18.68 | 20.09 |
|  | Clostridiales | 13.67 | 15.64 |
|  | Methanosarcinales | 11.40 | 2.21 |
|  | Bacillales | 5.19 | 4.48 |
|  | Thermotogales | 1.78 | 3.02 |
|  | Synergistales | 1.51 | 5.55 |
|  | Actinomycetales | 1.06 | 1.31 |
|  | Methanococcales | 1.03 | 0.45 |
|  | Desulfuromonadales | 0.97 | 1.01 |
|  | Halanaerobiales | 0.85 | 1.13 |
|  | Selenomonadales | 0.84 | 0.97 |
|  | Thermococcales | 0.67 | 0.72 |
|  | Methanomicrobiales | 0.65 | 0.40 |
|  | Lactobacillales | 0.60 | 0.65 |
|  | Rhizobiales | 0.57 | 0.54 |
|  | Chloroflexales | 0.55 | 0.47 |
|  | Burkholderiales | 0.55 | 0.47 |
|  | Desulfovibrionales | 0.53 | 0.60 |
|  | Chroococcales | 0.47 | 0.39 |
| Family | Methanobacteriaceae | 26.96 | 28.46 |
|  | Thermodesulfobiaceae | 11.72 | 12.09 |
|  | Methanosarcinaceae | 10.45 | 2.00 |
|  | Thermoanaerobacteraceae | 5.27 | 5.99 |
|  | Peptococcaceae | 4.50 | 5.05 |
|  | Clostridiaceae | 4.47 | 5.42 |
|  | Bacillaceae | 3.54 | 3.03 |
|  | Thermoanaerobacterales Family III. Incertae Sedis | 1.69 | 2.00 |
|  | Thermotogaceae | 1.68 | 2.85 |
|  | Synergistaceae | 1.51 | 5.55 |
|  | Syntrophomonadaceae | 1.42 | 1.46 |
|  | Methanothermaceae | 0.94 | 0.21 |
|  | Clostridiales Family XVIII. Incertae Sedis | 0.90 | 1.16 |
|  | Methanocaldococcaceae | 0.79 | 0.32 |
|  | Paenibacillaceae | 0.78 | 0.66 |
|  | Veillonellaceae | 0.76 | 0.86 |
|  | Clostridiales Family XVII. Incertae Sedis | 0.69 | 0.73 |
|  | Geobacteraceae | 0.67 | 0.68 |
|  | Thermococcaceae | 0.67 | 0.72 |
|  | Halanaerobiaceae | 0.59 | 0.89 |
|  | Chloroflexaceae | 0.52 | 0.43 |
|  | Alicyclobacillaceae | 0.51 | 0.45 |
|  | Unclassified (derived from Chroococcales) | 0.47 | 0.39 |
|  | Ruminococcaceae | 0.45 | 0.39 |
|  | Archaeoglobaceae | 0.45 | 0.42 |
|  | Heliobacteriaceae | 0.44 | 0.46 |
|  | Natranaerobiaceae | 0.42 | 0.34 |
|  | Desulfovibrionaceae | 0.40 | 0.47 |
|  | Nitrospiraceae | 0.36 | 0.25 |
|  | Thermaceae | 0.36 | 0.41 |
| Genus | *Methanothermobacter* | 24.35 | 25.84 |
|  | *Coprothermobacter* | 11.72 | 12.09 |
|  | *Methanosarcina* | 8.83 | 1.71 |
|  | *Clostridium* | 3.53 | 4.26 |
|  | *Bacillus* | 2.12 | 1.66 |
|  | *Caldanaerobacter* | 1.54 | 1.64 |
|  | *Moorella* | 1.39 | 1.65 |
|  | *Methanobrevibacter* | 1.30 | 0.23 |
|  | *Pelotomaculum* | 1.22 | 1.25 |
|  | *Desulfotomaculum* | 1.14 | 1.32 |
|  | *Methanohalophilus* | 1.11 | 1.21 |
|  | *Methanohalobium* | 0.97 | 0.96 |
|  | *Geobacillus* | 0.96 | 1.00 |
|  | *Carboxydothermus* | 0.95 | 1.15 |
|  | *Methanothermus* | 0.94 | 0.21 |
|  | *Alkaliphilus* | 0.94 | 1.16 |
|  | *Symbiobacterium* | 0.90 | 1.16 |
|  | *Thermoanaerobacter* | 0.86 | 0.99 |
|  | *Thermincola* | 0.83 | 0.97 |
|  | *Anaerobaculum* | 0.82 | 3.30 |
|  | *Desulfitobacterium* | 0.80 | 0.78 |
|  | *Methanocaldococcus* | 0.79 | 0.32 |
|  | *Caldicellulosiruptor* | 0.75 | 0.84 |
|  | *Thermotoga* | 0.71 | 1.13 |
|  | *Thermosediminibacter* | 0.70 | 0.86 |
|  | *Thermaerobacter* | 0.69 | 0.73 |
|  | *Geobacter* | 0.67 | 0.68 |
|  | *Paenibacillus* | 0.61 | 0.48 |
|  | *Ammonifex* | 0.53 | 0.56 |
|  | *Methanococcoides* | 0.53 | 0.44 |
| Species | *Methanothermobacter thermautotrophicus* | 14.80 | 15.53 |
|  | *Coprothermobacter proteolyticus* | 11.72 | 12.09 |
|  | *Methanosarcina barkeri* | 5.74 | 1.18 |
|  | *Methanothermobacter marburgensis* | 9.56 | 10.30 |
|  | *Methanosarcina mazei* | 3.09 | 0.53 |
|  | *Caldanaerobacter subterraneus* | 1.54 | 1.64 |
|  | *Moorella thermoacetica* | 1.39 | 1.65 |
|  | *Pelotomaculum thermopropionicum* | 1.22 | 1.25 |
|  | *Methanohalophilus mahii* | 1.11 | 1.21 |
|  | *Methanohalobium evestigatum* | 0.97 | 0.96 |
|  | *Carboxydothermus hydrogenoformans* | 0.95 | 1.15 |
|  | *Methanothermus fervidus* | 0.94 | 0.21 |
|  | *Symbiobacterium thermophilum* | 0.90 | 1.16 |
|  | *Clostridium thermocellum* | 0.83 | 1.16 |
|  | *Thermincola potens* | 0.83 | 0.97 |
|  | *Anaerobaculum hydrogeniformans* | 0.82 | 3.30 |
|  | *Desulfitobacterium hafniense* | 0.80 | 0.78 |
|  | *Methanobrevibacter smithii* | 0.76 | 0.13 |
|  | *Desulfotomaculum reducens* | 0.71 | 0.89 |
|  | *Thermosediminibacter oceani* | 0.70 | 0.86 |
|  | *Methanocaldococcus jannaschii* | 0.63 | 0.25 |
|  | *Alkaliphilus metalliredigens* | 0.60 | 0.69 |
|  | *Methanobrevibacter ruminantium* | 0.54 | 0.10 |
|  | *Ammonifex degensii* | 0.53 | 0.56 |
|  | *Methanococcoides burtonii* | 0.53 | 0.44 |
|  | *Candidatus Desulforudis audaxviator* | 0.52 | 0.73 |
|  | *Halothermothrix orenii* | 0.50 | 0.77 |
|  | *Dethiobacter alkaliphilus* | 0.50 | 0.40 |
|  | *Syntrophomonas wolfei* | 0.47 | 0.57 |
|  | *Candidatus Desulforudis* | 0.52 | 0.73 |

Note: MG: Metagenome; MT: Metatranscriptome; only top 20 at phylum, class and order level; top 30 at family, genus, species level is listed.

# Table S4. Contribution of the microbes to the overexpressed genes at the community and species level.

| **Genes** | | **KO**  **Entry** | **Community/**  **Microbes** | **Normalized**  **genes Abundance**  **(RPKs) *** | **Normalized**  **transcripts Abundance**  **(RPKs) *** | **Relative**  **Expression**  **value**** |
| --- | --- | --- | --- | --- | --- | --- |
| GroEL | Chaperonin GroEL | K04077 | Community | 2,170.7 | 1,467.5 | 38.3 |
|  |  |  | *Anaerobaculum hydrogeniformans* | 32.2 | 212.6 | 16.7 |
|  |  |  | *Anaerobaculum mobile* | 43.9 | 715.5 | 41.3 |
|  |  |  | *Coprothermobacter proteolyticus* | 1,492.8 | 173.9 | 0.3 |
|  |  |  | *Thermacetogenium phaeum* | 134.0 | 105.2 | 3.8 |
|  |  |  | Unclassified | 467.7 | 260.3 | 2,244.3 |
| DnaK | Molecular chaperone DnaK | K04043 | Community | 3,125.2 | 778.0 | 6.4 |
|  |  |  | *Anaerobaculum hydrogeniformans* | 38.6 | 34.2 | 2.2 |
|  |  |  | *Anaerobaculum mobile* | 13.4 | 9.6 | 1.8 |
|  |  |  | *Coprothermobacter proteolyticus* | 1,279.6 | 109.0 | 0.2 |
|  |  |  | *Methanothermobacter thermautotrophicus* | 116.9 | 1.4 | 0.0 |
|  |  |  | *Thermacetogenium phaeum* | 62.0 | 5.8 | 0.2 |
|  |  |  | Unclassified | 1,614.7 | 618.0 | 11.1 |
| CheY | Two-component system, chemotaxis family, response regulator CheY | K03413 | Community | 1,984.6 | 30,006.1 | 59.7 |
|  |  |  | *Anaerobaculum hydrogeniformans* | 44.4 | 9.0 | 0.5 |
|  |  |  | *Coprothermobacter proteolyticus* | 1,411.9 | 29,768.1 | 53.5 |
|  |  |  | *Thermacetogenium phaeum* | 227.9 | 76.6 | 7.1 |
|  |  |  | Unclassified | 300.4 | 152.5 | 6.4 |
| FlgE | Flagellar hook protein FlgE | K02390 | Community | 1,471.2 | 453.9 | 4.2 |
|  |  |  | *Anaerobaculum mobile* | 3.7 | 1.9 | 1.3 |
|  |  |  | *Coprothermobacter proteolyticus* | 1,412.3 | 438.3 | 0.8 |
|  |  |  | *Thermacetogenium phaeum* | 7.0 | 8.0 | 2.9 |
|  |  |  | Unclassified | 48.1 | 5.7 | 0.4 |
| FlgB | Flagellar basal-body rod protein FlgB | K02387 | Community | 1,318.6 | 425.0 | 1.0 |
|  |  |  | *Coprothermobacter proteolyticus* | 1,184.9 | 402.3 | 0.9 |
|  |  |  | *Thermacetogenium phaeum* | 67.2 | 0.1 | 0.0 |
| FliF | Flagellar M-ring protein FliF | K02409 | Community | 1,408.0 | 103.0 | 1.4 |
|  |  |  | *Anaerobaculum mobile* | 14.5 | 8.0 | 1.4 |
|  |  |  | *Coprothermobacter proteolyticus* | 1,333.9 | 89.2 | 0.2 |
|  |  |  | *Thermacetogenium phaeum* | 44.0 | 5.5 | 0.3 |
|  |  |  | Unclassified | 15.6 | 0.3 | 0.1 |

Note: * The abundance of genes and responding transcripts is normalized by the gene length and sequencing depth. RPKs: reads per kilobases;

** The relative expression value of genes is the normalized abundance value of cDNA divided by the normalized abundance value of DNA and adjusted for differences in sequencing depth.

# Table S6. Detailed information of the functional enzymes involved in the methanogenesis pathways.

| Enzymes  (Genes) | Involved Enzymes | Involved Pathway | Definition | EC  Number | Hits  (MG) | Hits  (MT) | Percent  (MG, ‰) | Percent  (MT, ‰) | MT/MG |
| --- | --- | --- | --- | --- | --- | --- | --- | --- | --- |
| Fdh | fdhA,FDH | HA/SAO/  HM | formaldehyde dehydrogenase | 1.2.1.46/  1.2.1.2 | 297 | 5 | 0.1228 | 0.0271 | 0.22 |
| Fhs  (FTHFS) | fhs | HA/SAO | formate tetrahydrofolate ligase | 6.3.4.3 | 4431 | 1220 | 1.8325 | 6.6081 | 3.61 |
| FolD/  MTHFD | folD/MTHFD/MTHFD1L/  MTHFD2 | HA/SAO | methylenetetrahydrofolate dehydrogenase (NADP+)/methenyltetrahydrofolate cyclohydrolase/formyltetrahydrofolate synthetase | 1.5.1.5/  3.5.4.9/  6.3.4.3 | 46 | 7 | 0.0190 | 0.0379 | 1.99 |
| MetF | metF | HA/SAO | methylenetetrahydrofolate reductase (NADPH) | 1.5.1.20 | 1379 | 199 | 0.5703 | 1.0779 | 1.89 |
| Mtr | mtrA-H | HA/SAO/  HM/AM | tetrahydromethanopterin S-methyltransferase subunit A-H | 2.1.1.86 | 13528 | 1838 | 5.5947 | 9.9554 | 1.78 |
| CODH/  ACS | CODH-ACSA/  CODH-ACSB | HA/SAO/AM | carbon monoxide dehydrogenase/acetyl-CoA synthase subunit alpha/acetyl-CoA synthase subunit beta | 1.2.7.4/  1.2.99.2/  2.3.1.169 | 1537 | 899 | 0.6356 | 4.8694 | 7.66 |
| Pta | pta | HA/SAO/AM | phosphate acetyltransferase | 2.3.1.8 | 1860 | 950 | 0.7692 | 5.1456 | 6.69 |
| Ack | ackA | HA/SAO/AM | acetate kinase | 2.7.2.1 | 4677 | 1505 | 1.9342 | 8.1517 | 4.21 |
| Fwd/Fmd | fwdA-D,F-H/  fmdA-F | HM | formylmethanofuran dehydrogenase subunit A-H | 1.2.99.5 | 20023 | 699 | 8.2807 | 3.7861 | 0.46 |
| Ftr | ftr | HM | formylmethanofuran tetrahydromethanopterin N-formyltransferase | 2.3.1.101 | 2983 | 98 | 1.2337 | 0.5308 | 0.43 |
| Mch | mch | HM | methenyltetrahydromethanopterin cyclohydrolase | 3.5.4.27 | 2225 | 51 | 0.9202 | 0.2762 | 0.30 |
| Mtd/Hmd | mtd/hmd | HM | methylenetetrahydromethanopterin dehydrogenase | 1.5.99.9/  1.12.98. | 3879 | 284 | 1.6042 | 1.5383 | 0.96 |
| Mer | mer | HM | coenzyme F420-dependent N5,N10-methenyltetrahydromethanopterin reductase | 1.5.99.11 | 2360 | 435 | 0.9760 | 2.3562 | 2.41 |
| Mcr | mcrA-D,G | HM/AM | methyl-coenzyme M reductase alpha/beta/gamma/C/D subunit | 2.8.4.1 | 11971 | 7645 | 4.9507 | 41.4087 | 8.36 |
| Hdr/Mvh/  Vho/ Vhu/Vhc | hdrA-E/  mvhA,D,G/  vhuA,D,G/  vhcA,D,G/  vhoA,C,G | HM/AM | heterodisulfide reductase subunit A-E/  F420-non-reducing hydrogenase subunit A,D,G/F420-nonreducing hydrogenase I | 1.8.98.1/  1.12.99.-/  1.12.2.- | 22860 | 2028 | 9.4540 | 10.9845 | 1.16 |
| Frh | frhA/B/D/G | HM | coenzyme F420 hydrogenase alpha/beta/delta/gamma subunit | 1.12.98.1 | 10523 | 536 | 4.3519 | 2.9032 | 0.67 |
| Nha | nhaA,C | HM | Na+:H+ antiporter, NhaA family/NhaC family | / | 362 | 197 | 0.1497 | 1.0670 | 7.13 |
| Eha/Ehb | ehaA-O/  ehbA-Q | HM | energy-converting hydrogenase A subunit A-O/  energy-converting hydrogenase B subunit A-Q | / | 6753 | 53 | 2.7928 | 0.2871 | 0.10 |
| Ech | echA-F | AM | ech hydrogenase subunit A-F | / | 3228 | 660 | 1.3350 | 3.5749 | 2.68 |
| Cdh | cdhA-E | AM | acetyl-CoA decarbonylase/synthase complex subunit A-E | 1.2.99.2/  2.3.1.-/  2.1.1.245 | 10627 | 2995 | 4.3949 | 16.2222 | 3.69 |
| ATPase | atpA-H/  ntpA-F,I,K | HA/SAO/  HM/AM | H+-transporting ATPase/  K+-transporting ATPase/  Ca2+ transporting ATPase/  transitional endoplasmic reticulum ATPase | 3.6.4.6/  3.6.4.9/  3.6.4.10/  3.6.4.12/  3.6.4.14 | 59109 | 6208 | 24.4452 | 33.6253 | 1.38 |

Note: “MT/MG” indicates the ratios of the relative abundances of these different genes from the metatranscriptome and metagenome; HA: homoacetogenesis; HM: hydrogenophilic methanogenesis; SAO: syntrophic acetate oxidation; AM: acetoclastic methanogenesis.
